# Plate-like Guanine Biocrystals Form via Templated Nucleation of Crystal Leaflets on Preassembled Scaffolds

**DOI:** 10.1021/jacs.2c11136

**Published:** 2022-12-05

**Authors:** Zohar Eyal, Rachael Deis, Neta Varsano, Nili Dezorella, Katya Rechav, Lothar Houben, Dvir Gur

**Affiliations:** † Department of Molecular Genetics, Weizmann Institute of Science, Rehovot 76100, Israel; ‡ Department of Chemical Research Support, Weizmann Institute of Science, Rehovot 76100, Israel

## Abstract

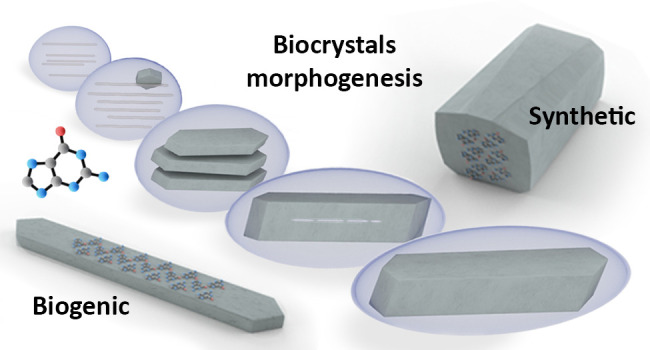

Controlling the morphology of crystalline materials is challenging, as crystals have a strong tendency toward thermodynamically stable structures. Yet, organisms form crystals with distinct morphologies, such as the plate-like guanine crystals produced by many terrestrial and aquatic species for light manipulation. Regulation of crystal morphogenesis was hypothesized to entail physical growth restriction by the surrounding membrane, combined with fine-tuned interactions between organic molecules and the growing crystal. Using cryo-electron tomography of developing zebrafish larvae, we found that guanine crystals form via templated nucleation of thin leaflets on preassembled scaffolds made of 20-nm-thick amyloid fibers. These leaflets then merge and coalesce into a single plate-like crystal. Our findings shed light on the biological regulation of crystal morphogenesis, which determines their optical properties.

The nanoscale morphologies of crystalline materials determine their optical, electrical, and mechanical properties and, thus, their potential application.^1−5^ In nature, many organisms use molecular crystals for their function, which they form out of small organic molecules under ambient conditions.^6−10^ A prominent example is the thin plate-like guanine crystals, utilized by a huge variety of terrestrial and aquatic organisms for diverse functions such as vision, camouflage, body temperature regulation, and kin recognition.^6,7,10−13^ Guanine crystals are constructed from H-bonded molecular layers that are stacked one on top of the other by π-stacking.^14^ Plate-like guanine crystals expose the extremely high, in-plane refractive index (*n* = 1.83) to light, thereby allowing the construction of highly effective and versatile photonic arrays (Figure 1A, B).^15^ However, the inherent thermodynamic properties of the crystalline lattice create a strong tendency toward forming specific low-energy prismatic structures.^7,16,17^ Overriding this intrinsic tendency of the crystals to grow as prisms requires extensive biological intervention to selectively impede the growth along the π-stacking direction (Figure 1A, B). Yet, the mechanism that exerts such tight and extensive control over crystal morphology has remained a mystery.

**Figure 1:**
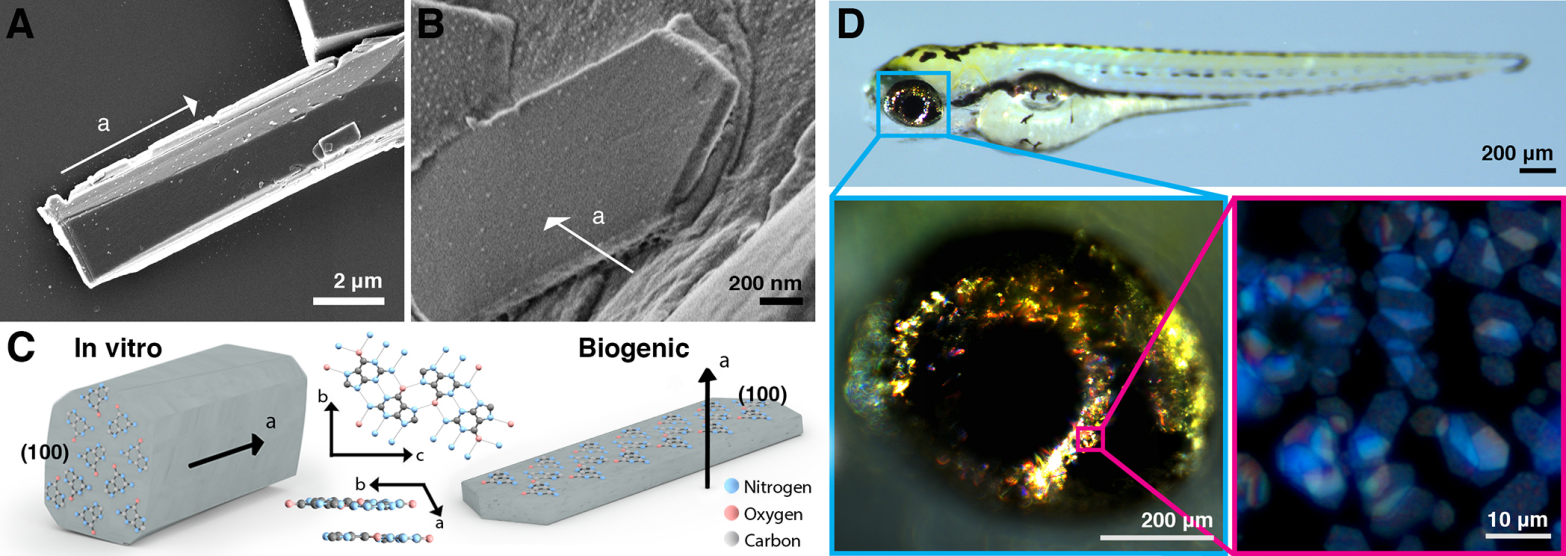
Synthetic and biogenic guanine crystals have distinct morphologies. (A) A synthetic guanine crystal with a prismatic morphology, where the (100) crystallographic plane is the fastest growing direction. (B) A biogenic plate-like guanine crystal in a zebrafish eye. (C) Schematic illustration of both synthetic and biogenic crystals, where the (100) crystallographic plane is constructed from H-bonded molecular layers. (D) A zebrafish larva at 5 days post-fertilization contains guanine crystals in its eyes and skin. Insets show higher magnifications of the eye (cyan) and crystal-containing iridophores (pink). A and B are SEM micrographs and D shows incident light images.

Specialized guanine crystal-forming cells, also known as iridophores, produce crystals within membrane-bound organelles termed iridosomes,^18,19^ where exquisite control over crystal size, shape, and assembly far exceeds the synthetic state-of-the-art in materials science and solid-state chemistry.^7,20^ Studies on melanophores, neural crest derived pigment cells,^21^ indicate that their melanin-producing organelles, called melanosomes, are derived from endosomal compartments and belong to the lysosome-related organelles (LROs) family.^22^ However, much less is known about non-melanosomal pigment organelles such as the iridosome.^23−25^ Over the years, several mechanisms that control biocrystal morphogenesis have been suggested, including the involvement of stereochemical interactions with different biomolecules.^9,26,27^ These molecules were postulated to interact with the growing crystals stereochemically and thereby create local kinetic conditions that allow for the formation of diverse crystal morphologies. However, studies of the biochemical composition of guanine crystals with different morphologies from a variety of organisms suggest that the small molecule composition of crystals does not correlate with their shape.^28^ In spiders, prismatic crystals were proposed to form on sheets within a crystal vesicle^29,30^ and to comprise 25-nm-thick crystal lamellas stacks.^30^ Studies on inorganic biocrystals suggest that crystal morphogenesis is controlled by physical confinement by the delimiting membrane of the organelle,^31^ which allows for growth rate manipulations that are sufficient to produce a variety of complex shapes.

To elucidate the morphogenesis of guanine plate-like crystals and the underlying mechanism, we investigated iridophores in the zebrafish (*Danio rerio*) model organism, which uses guanine crystals in their eyes both as a light barrier and to enhance vision sensitivity under low light conditions (Figure 1C).^32,33^ Synchronized crystal growth in the zebrafish larva iridophores starts as early as 44 h post-fertilization (hpf), making it an ideal system to follow the early stages of crystal formation.^34^ To study the early events of crystal formation in zebrafish larvae eyes (Figure 1D) in their native hydrated state, we used cryogenic scanning electron microscopy (cryo-SEM) (Figure 2A-D) to image iridophores adjacent to melanophores in the retina (Figure 2A-C). In adult fish, mature iridophores are packed with dozens of membrane-bound, plate-like crystals (Figure S1). At earlier developmental stages, the plate-like crystals were composed of very thin leaflets, a few nanometers in thickness, representing only 5–20 molecular stacks of guanine sheets (Figure 2D). During subsequent maturation, the leaflets expanded and eventually coalesced into a single crystal (Figure 2D, last panel).

**Figure 2:**
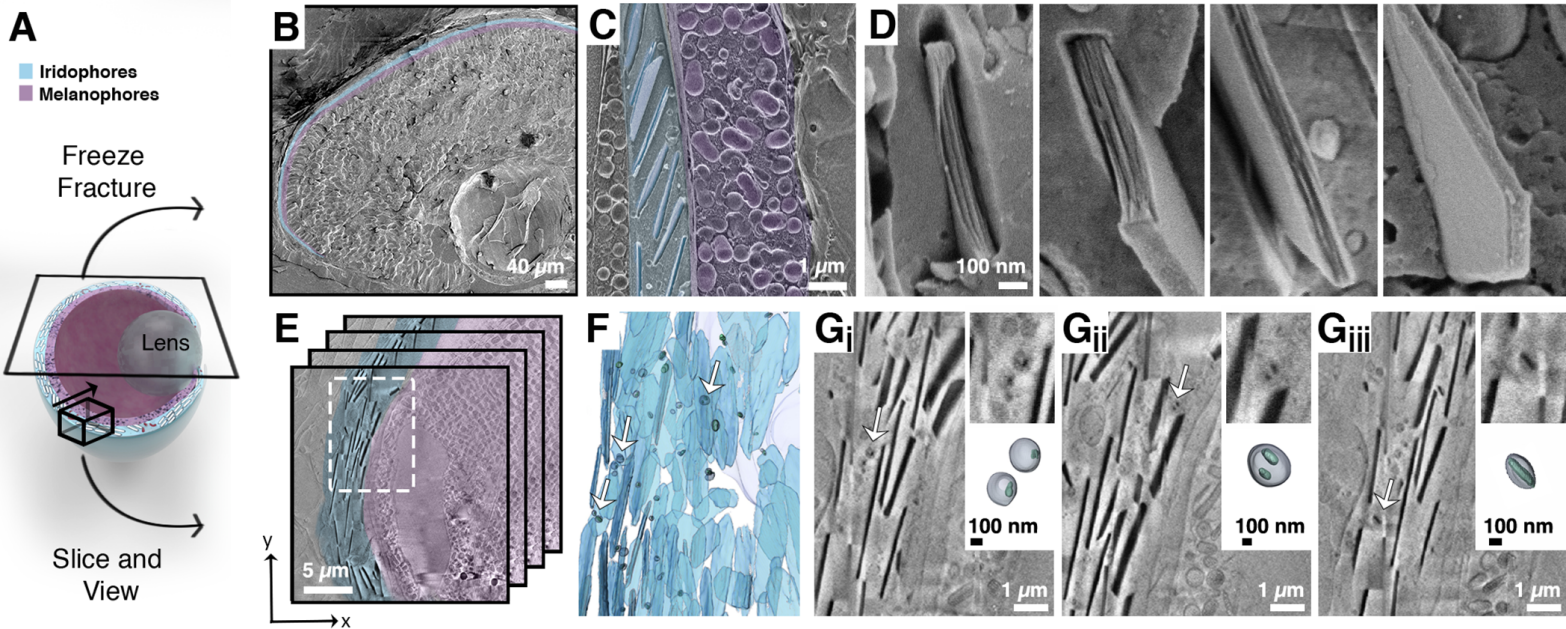
Early iridosomes contain several crystal leaflets that later coalesce into a single plate-like crystal. (A) An illustration of the zebrafish larva eye showing the iridophore (blue) and melanophore layers (purple), next to the lens. The anatomical location of the sections and areas that were taken for cryo-SEM (B-D) and cryo-FIB-SEM (E-G) are marked by a black rectangle and a black box, respectively. (B-D) Cryo-SEM images of freeze-fractured surfaces of a zebrafish larva eye and the iridophores within it. (B) A low magnification of the eye surface. Iridophore and melanophore layers are pseudocolored in blue and purple, respectively. (C) A close-up view of an iridophore next to a melanophore. (D) Iridosomes at different maturation stages. (E-G) Cryo-FIB-SEM images and 3D representations of segmented milled sections from a freeze-fractured zebrafish larva eye. (E) An illustration showing the stack of images of consecutive volumetric sections, with pseudocolored iridophore and melanophore layers. (F) An eye iridophore 3D representation of the area marked by a dashed rectangle in E. Mature elongated iridosomes and round developing iridosomes are marked in blue, nucleus is marked in gray. The early 200–300-nm-long iridosomes shown in (G_i_-G_iii_) are marked with white arrows. (G_i_-G_iii_) Cryo-FIB-SEM micrographs of different planes within the iridophore shown in (F). White arrows mark the iridosomes whose 3D reconstructions are shown in the insets.

Capturing the initial transient stages of crystal formation is challenging and requires obtaining 3D volumetric data, which allows for the examination of the entire volume of the iridosomes in their native state. Therefore, we used cryogenic focused ion beam SEM imaging (cryo-FIB-SEM) to study early iridophores *in situ*, inside the zebrafish larvae eyes (Figure 2E-G). In cryo-FIB-SEM, guanine crystals appear as dark contrast objects, due to their surface potential. Using this approach, we found small, ∼150–300 nm round iridosomes next to more mature, elongated iridosomes, which were already several micrometers in length (Figure 2F, G and Movie S1). Surprisingly, the small and round iridosomes contained thin, well-developed crystals (Figure 2F, G and Figure S2). As crystal growth advanced, iridosomes gradually acquired the typical elliptical shape, where the membrane tightly engulfs the elongated crystal. In the smallest and most immature iridosomes, we observed distinct thin leaflets that did not appear to fill the entire volume of the iridosome. In certain cases, multiple thin leaflets were present in the same iridosome (Figure 2G).

Since the early stages of crystal formation occur at the nanometric scale, they are barely visible using cryo-SEM and cryo-FIB-SEM modalities. Thus, we utilized high-resolution cryo-electron tomography (cryoET)^35^ to investigate crystal formation in 3D (Figure 3). Notably, in this approach the intact membranes are clearly visible, which allows studying the interface between the membrane and developing crystal and the role of confinement in this process. To image iridosomes from different developmental stages, we plunge-freezed iridophores that were isolated from *pnp4a:palmmCherry*(36) transgenic zebrafish larvae using fluorescence-activated cell sorting (FACS)^37^ (Figure S3). In iridophores that were isolated from ∼72 hpf larvae, we observed two main types of crystal organization. Crystals were positioned either with their (100) facet face-on (Figure 3A, D, G) or edge-on (Figure 3C, E, F, H) with respect to the electron beam. In all observed iridosomes, the initial crystals formed within the lumen of the organelle with no apparent contact with the surrounding bilayer membrane (Figure 3A-C and Movie S2). The elongated crystals then continued to grow toward the surrounding membrane until they eventually made contact, and started pushing against it (Figure 3D). At this point, additional crystal growth resulted in deformation and elongation of the surrounding membrane (Figure 3G), until both crystal and membrane assumed their final elongated morphology (Figure S4).

**Figure 3:**
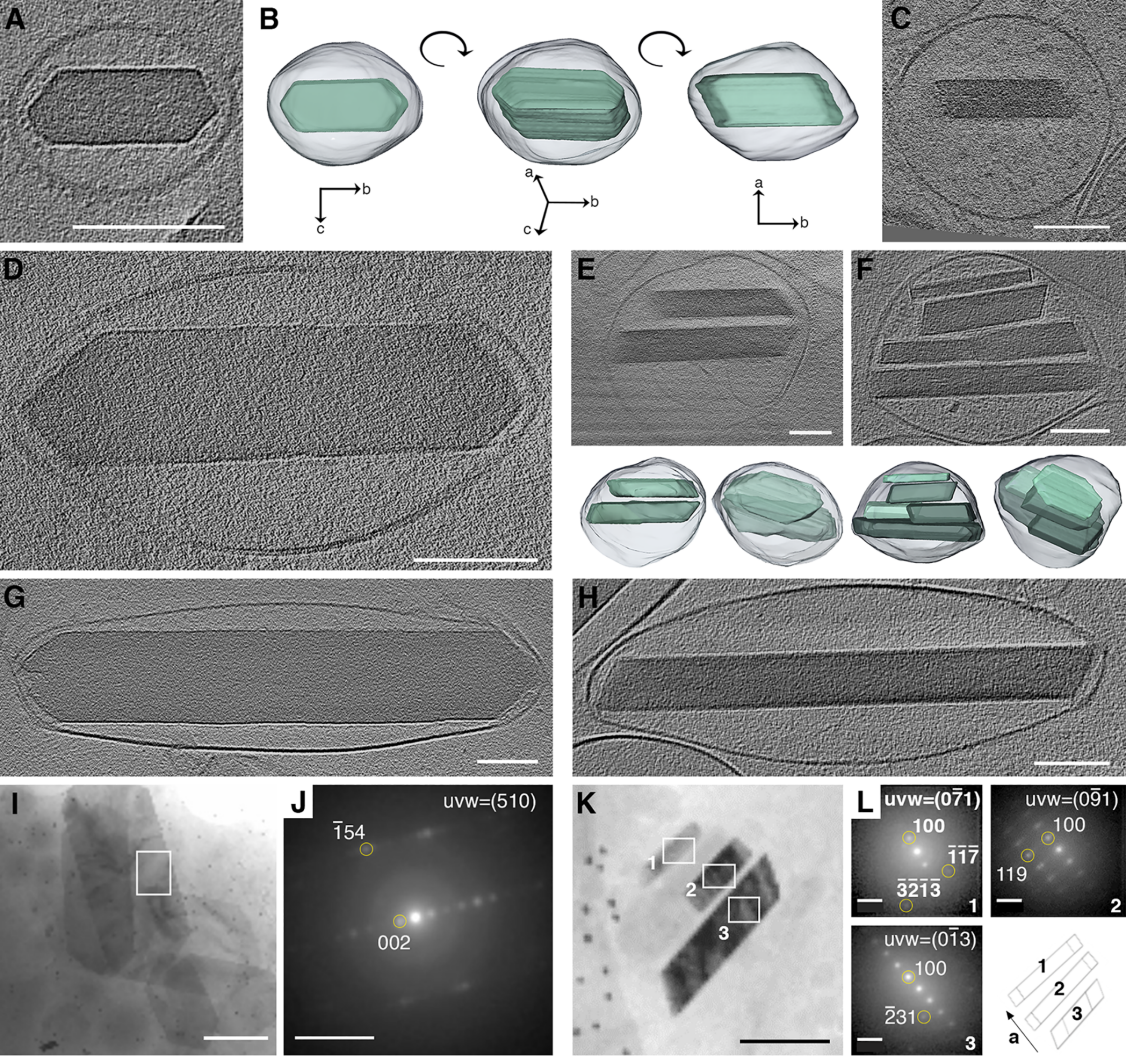
High-resolution cryo-TEM shows the morphological sequence of (100) crystal plates within iridosomes. (A, C–H) CryoET reconstructions of iridophores, isolated from zebrafish larvae at different developmental stages, show crystals either face-on (A,D,G) or edge-on (C,E,F,H) with respect to the electron beam. (B) A 3D representation of a crystal-containing iridosome from different angles. Bottom panels in (E) and (F) show 3D representations of leaflet-containing iridosomes. (I,K) Cryo-4D-STEM bright field images of iridosomes with face-on (I) or edge-on (K) orientated crystals. (J) Electron diffractions taken from the area marked by a white rectangle in (I). (L) Electron diffractions taken from the area marked by a white rectangle in (K), and an illustration showing the orientation of the *a* axes of the crystals (bottom right corner). A-I, K, scale bars are 100 nm. J, L, scale bars are 5 nm^–1^.

In iridosomes where the plate-like crystals were orientated edge-on, we often observed 2–8 distinct crystal leaflets within the same organelle (Figure 3E-F and Movie S3). To correlate the morphological information with crystallographic features, we investigated iridosomes using cryogenic 4D scanning transmission electron microscopy (cryo-4D-STEM) to collect an electron diffraction pattern from every point the beam raster traverses (Figure 3I-L). We found that crystal leaflets that were oriented face-on exhibited a diffraction pattern that correlated to the (002) crystallographic plane (Figure 3I-J), whereas in leaflets that assumed the perpendicular orientation, it correlated to the (100) crystallographic plane (Figure 3K, L). These results strongly indicate that the very initial leaflets are already formed as (100) crystal plates. In developing iridosomes that contained multiple crystal leaflets, we often observed that the leaflets were not fully aligned (Figure 3E-F, Figure S4). However, when we used cryo-4D-STEM to map the orientation of crystals in more developed iridosomes, we found that the crystal leaflets were arranged such that their *a* axes were almost completely parallel (Figure 3K, L). Remarkably, in mature iridosomes, the leaflets coalesced into a single crystal with no obvious remnants of the initial leaflets.

To investigate early nucleation events during crystal leaflet formation, we isolated iridophores from younger zebrafish larvae (44–48 hpf) (Figure 4). Intriguingly, we found that the iridosomes in these very early cells contained up to 10 usually parallel fibers running across them. The fibers were approximately 20 nm in diameter and 200–400 nm in length. Similarly, the smallest crystals observed were approximately 20 nm in size (Figure 4A and Figure S5). Imaging of more developed iridosomes revealed small crystals in close contact with the preassembled fiber scaffold (Figure 4B). Remarkably, these initial crystals already formed as thin leaflets, assuming the typical (100) plate-like morphology. Staining with Thioflavin T (ThT), a well-established amyloid marker,^38^ indicated that these fiber scaffolds are proteins, aggregated as amyloid fibers (Figure S6). Fast Fourier transform (FFT) analysis on images of fiber-containing iridosomes revealed that the fibers had a periodicity of ∼1.9 nm (Figure S5), corresponding to the inter ribbon spacing of the β-sheet fibril.^39^

**Figure 4:**
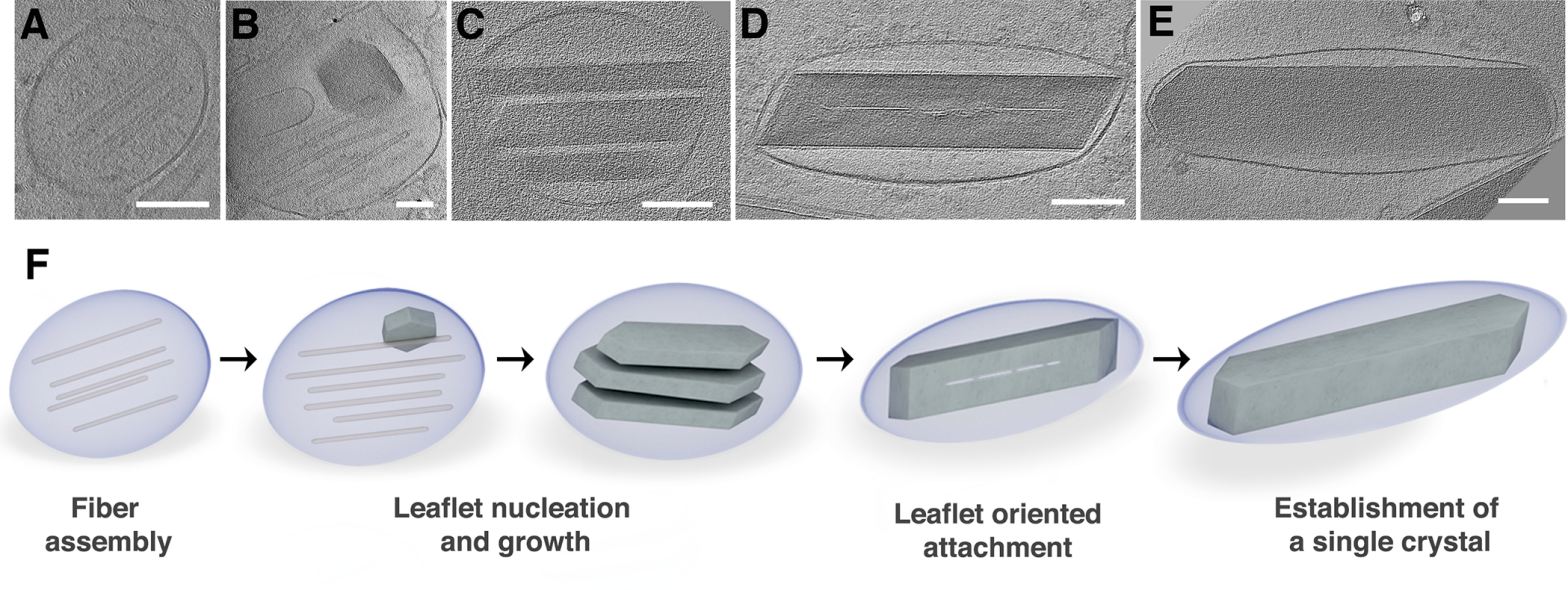
Biogenic plate-like guanine crystals form via templated nucleation of (100) crystal leaflets on preassembled protein fibers. (A-E) CryoET reconstructions of iridophores isolated from zebrafish larvae at different maturation stages. Scale bars are 100 nm. (A) An early iridosome showing a scaffold of parallel protein fibers. (B) A more developed iridosome, in which the initial nucleation of (100) crystal leaflet on a protein scaffold is taking place. (C) Several individual leaflets, imaged edge-on, with their (100) crystal plane parallel to each other. (D) The leaflets have almost completely coalesced into a single crystal. (E) A mature iridosome, where the leaflets have completely merged into a single crystal. (F) Schematic illustration of the proposed crystallization mechanism.

The formation of thin plate-like guanine crystals requires overriding the intrinsic tendency of crystals to grow as prisms and to preferentially express the thermodynamically disfavored (100) face.^14^ Although recent studies suggest that the control over morphology of certain biocrystals involves confinement,^31^ we found that initial crystal formation occurs at a considerable distance from the delimiting membrane. Only later, the elongating crystals reach the membrane and push against it. Based on our high-resolution cryoET of iridophores at different developmental stages, we propose that the formation of plate-like crystals occurs via templated nucleation of thin leaflets on preassembled amyloid fiber scaffolds. This process begins with the assembly of the protein scaffolds inside the iridosome (Figure 4A). Then, nucleation of distinct (100) crystal plates takes place on the fibers (Figure 4B). As development advances, the number and size of crystal leaflets increases (Figure 4C), followed by their alignment and gradual oriented attachment (Figure 4D), culminating in the formation of a single plate-like guanine crystal (Figure 4E). As crystal formation progressed, we no longer detected the fibers. This could be due to the incorporation of the fibers in the crystal lattice or their disassembly due to the changing iridosome microenvironment. Figure 4F schematically illustrates this sequence of events. Because of the transient nature of early stages of crystal nucleation, identifying the exact nucleation spot is extremely challenging. Yet, the preassembled amyloid fiber scaffolds likely drive crystal growth along the H-bonded direction through molecular recognition, either at the level of the basic building blocks, i.e., interactions between specific amino acids and guanine molecules, or at a macromolecular level, by promoting the interaction of hydrophobic domains in the fiber with the planar sheets of H-bonded guanine layers. In melanosomes, it was shown that fibrillar sheets of PMEL, a nonpathogenic amyloid protein, serve as a template for melanin polymerization and synthesis and that lack of PMEL results in different degrees of hypopigmentation.^40,41^ Identifying the amyloid protein and uncovering its structure could further elucidate the mechanism of controlled crystal formation, shedding light on the interactions between proteins and molecular crystals and on the mechanisms that control crystal nucleation and growth.

## Supporting Information Available

Movie S1 show cryo-FIB-SEM 3D volume rendering data of iridophores within a zebrafish larvae eye (AVI)
Movie S2 show cryoET 3D volume rendering data of an iridosome (MOV)
Movie S3 show cryoET 3D volume rendering of an early iridosome (MPG)
Methods section and figures, including sample preparation and data collection techniques (PDF)

## Supplementary Material

ja2c11136_si_001.avi

ja2c11136_si_002.mov

ja2c11136_si_003.mpg

## Abbreviations

LRO: lysosome-related organelles
hpf: hours post-fertilization
cryo-SEM: cryogenic scanning electron microscopy
cryo-FIB-SEM: cryogenic focused ion beam SEM
cryoET: cryo-electron tomography
ThT: Thioflavin T
FFT: fast Fourier transform

## References

[ref1] Lowenstam H. A.; Weiner S. On biomineralization; Oxford University Press, 1989.

[ref2] Aizenberg J.; Tkachenko A.; Weiner S.; Addadi L.; Hendler G. Calcitic microlenses as part of the photoreceptor system in brittlestars. Nature 2001, 412 (6849), 819–822. 10.1038/35090573. 11518966

[ref3] De Yoreo J. J.; Vekilov P. G. Principles of Crystal Nucleation and Growth. Rev. Mineral Geochem. 2003, 54 (1), 57–93. 10.2113/0540057.

[ref4] Weiner S.; Dove P. M. An overview of biomineralization processes and the problem of the vital effect. Rev. Mineral Geochem 2003, 54 (1), 1–29. 10.2113/0540001.

[ref5] Tadayon M.; Younes-Metzler O.; Shelef Y.; Zaslansky P.; Rechels A.; Berner A.; Zolotoyabko E.; Barth F. G.; Fratzl P.; Bar-On B.; Politi Y. Adaptations for Wear Resistance and Damage Resilience: Micromechanics of Spider Cuticular ″Tools″. Adv. Funct. Mater. 2020, 30 (32), 2000400 10.1002/adfm.202000400.

[ref6] Land M. The physics and biology of animal reflectors. Prog. Biophys. Mol. Biol. 1972, 24, 75–106. 10.1016/0079-6107(72)90004-1. 4581858

[ref7] Gur D.; Palmer B. A.; Weiner S.; Addadi L. Light Manipulation by Guanine Crystals in Organisms: Biogenic Scatterers, Mirrors, Multilayer Reflectors and Photonic Crystals. Adv. Funct. Mater. 2017, 27 (6), 1603514 10.1002/adfm.201603514.

[ref8] Palmer B. A.; Gur D.; Weiner S.; Addadi L.; Oron D. The Organic Crystalline Materials of Vision: Structure-Function Considerations from the Nanometer to the Millimeter Scale. Adv. Mater. 2018, 30 (41), 1800006 10.1002/adma.201800006. 29888511

[ref9] Weiner S.; Addadi L. Crystallization Pathways in Biomineralization. Annu. Rev. Mater. Res. 2011, 41, 21–40. 10.1146/annurev-matsci-062910-095803.

[ref10] Jantschke A.; Pinkas I.; Hirsch A.; Elad N.; Schertel A.; Addadi L.; Weiner S. Anhydrous beta-guanine crystals in a marine dinoflagellate: Structure and suggested function. J. Struct. Biol. 2019, 207 (1), 12–20. 10.1016/j.jsb.2019.04.009. 30991101

[ref11] Levy-Lior A.; Shimoni E.; Schwartz O.; Gavish-Regev E.; Oron D.; Oxford G.; Weiner S.; Addadi L. Guanine-Based Biogenic Photonic-Crystal Arrays in Fish and Spiders. Adv. Funct. Mater. 2010, 20 (2), 320–329. 10.1002/adfm.200901437.

[ref12] Gur D.; Leshem B.; Oron D.; Weiner S.; Addadi L. The Structural Basis for Enhanced Silver Reflectance in Koi Fish Scale and Skin. J. Am. Chem. Soc. 2014, 136 (49), 17236–17242. 10.1021/ja509340c. 25393507

[ref13] Gur D.; Leshem B.; Pierantoni M.; Farstey V.; Oron D.; Weiner S.; Addadi L. Structural Basis for the Brilliant Colors of the Sapphirinid Copepods. J. Am. Chem. Soc. 2015, 137 (26), 8408–11. 10.1021/jacs.5b05289. 26098960

[ref14] Hirsch A.; Gur D.; Polishchuk I.; Levy D.; Pokroy B.; Cruz-Cabeza A. J.; Addadi L.; Kronik L.; Leiserowitz L. Guanigma”: The Revised Structure of Biogenic Anhydrous Guanine. Chem. Mater. 2015, 27 (24), 8289–8297. 10.1021/acs.chemmater.5b03549.

[ref15] Levy-Lior A.; Pokroy B.; Levavi-Sivan B.; Leiserowitz L.; Weiner S.; Addadi L. Biogenic guanine crystals from the skin of fish may be designed to enhance light reflectance. Cryst. Growth Des. 2008, 8 (2), 507–511. 10.1021/cg0704753.

[ref16] Gur D.; Pierantoni M.; Elool Dov N.; Hirsh A.; Feldman Y.; Weiner S.; Addadi L. Guanine Crystallization in Aqueous Solutions Enables Control over Crystal Size and Polymorphism. Cryst. Growth Des. 2016, 16 (9), 4975–4980. 10.1021/acs.cgd.6b00566.

[ref17] Wittig N. K.; Christensen T. E. K.; Grunewald T. A.; Birkedal H. Vase-like beta-Polymorph Guanine Crystal Aggregates Formed at the Air-Water Interface. ACS Mater. Lett. 2020, 2 (5), 446–452. 10.1021/acsmaterialslett.9b00447.

[ref18] Ballowitz E. Über chromatische Organe in der Haut von Knochenfischen. Anat. Anz 1912, 42, 186–190.

[ref19] Odiorne J. M. The Occurrence of Guanophores in Fundulus. Proc. Natl. Acad. Sci. U. S. A. 1933, 19 (7), 750–4. 10.1073/pnas.19.7.750. 16577560 PMC1086146

[ref20] Corpinot M. K.; Bucar D. K. A Practical Guide to the Design of Molecular Crystals. Cryst. Growth Des. 2019, 19 (2), 1426–1453. 10.1021/acs.cgd.8b00972.

[ref21] Rawles M. E. Origin of melanophores and their role in development of color patterns in vertebrates. Physiol. Rev. 1948, 28 (4), 383–408. 10.1152/physrev.1948.28.4.383. 18894955

[ref22] Bowman S. L.; Bi-Karchin J.; Le L.; Marks M. S. The road to lysosome-related organelles: Insights from Hermansky-Pudlak syndrome and other rare diseases. Traffic 2019, 20 (6), 404–435. 10.1111/tra.12646. 30945407 PMC6541516

[ref23] Figon F.; Hurbain I.; Heiligenstein X.; Trepout S.; Lanoue A.; Medjoubi K.; Somogyi A.; Delevoye C.; Raposo G.; Casas J. Catabolism of lysosome-related organelles in color-changing spiders supports intracellular turnover of pigments. Proc. Natl. Acad. Sci. U. S. A. 2021, 118 (35), e2103020118 10.1073/pnas.2103020118. 34433668 PMC8536372

[ref24] Ullate-Agote A.; Burgelin I.; Debry A.; Langrez C.; Montange F.; Peraldi R.; Daraspe J.; Kaessmann H.; Milinkovitch M. C.; Tzika A. C. Genome mapping of a LYST mutation in corn snakes indicates that vertebrate chromatophore vesicles are lysosome-related organelles. Proc. Natl. Acad. Sci. U. S. A. 2020, 117 (42), 26307–26317. 10.1073/pnas.2003724117. 33020272 PMC7584913

[ref25] Figon F.; Deravi L. F.; Casas J. Barriers and Promises of the Developing Pigment Organelle Field. Integr. Comp. Biol. 2021, 61 (4), 1481–1489. 10.1093/icb/icab164. 34283212

[ref26] Belcher A. M.; Wu X. H.; Christensen R. J.; Hansma P. K.; Stucky G. D.; Morse D. E. Control of crystal phase switching and orientation by soluble mollusc-shell proteins. Nature 1996, 381 (6577), 56–58. 10.1038/381056a0.

[ref27] Shtukenberg A. G.; Ward M. D.; Kahr B. Crystal Growth with Macromolecular Additives. Chem. Rev. 2017, 117 (24), 14042–14090. 10.1021/acs.chemrev.7b00285. 29165999

[ref28] Pinsk N.; Wagner A.; Cohen L.; Smalley C. J. H.; Hughes C. E.; Zhang G.; Pavan M. J.; Casati N.; Jantschke A.; Goobes G.; Harris K. D. M.; Palmer B. A. Biogenic Guanine Crystals Are Solid Solutions of Guanine and OtherPurine Metabolites. J. Am. Chem. Soc. 2022, 144 (11), 5180–5189. 10.1021/jacs.2c00724. 35255213 PMC8949762

[ref29] Seitz K. A. Elektronenmikroskopische untersuchungen an den Guanin-Speicherzellen von Araneus diadematus clerck (Araneae, Araneidae). Z. Morphol. Tiere 1972, 72 (3), 245–262. 10.1007/BF00391554.

[ref30] Wagner A.; Ezersky V.; Maria R.; Upcher A.; Lemcoff T.; Aflalo E. D.; Lubin Y.; Palmer B. A. The Non-Classical Crystallization Mechanism of a Composite Biogenic Guanine Crystal. Adv. Mater. 2022, 34 (31), 2202242 10.1002/adma.202202242. 35608485

[ref31] Avrahami E. M.; Houben L.; Aram L.; Gal A. Complex morphologies of biogenic crystals emerge from anisotropic growth of symmetry-related facets. Science 2022, 376 (6590), 312–316. 10.1126/science.abm1748. 35420932

[ref32] Hirata M.; Nakamura K.; Kondo S. Pigment cell distributions in different tissues of the zebrafish, with special reference to the striped pigment pattern. Developmental dynamics: an official publication of the American Association of Anatomists 2005, 234 (2), 293–300. 10.1002/dvdy.20513. 16110504

[ref33] Gur D.; Nicolas J. D.; Brumfeld V.; Bar-Elli O.; Oron D.; Levkowitz G. The Dual Functional Reflecting Iris of the Zebrafish. Adv. Sci. 2018, 5 (8), 1800338 10.1002/advs.201800338. PMC6097150 30128243

[ref34] Petratou K.; Subkhankulova T.; Lister J. A.; Rocco A.; Schwetlick H.; Kelsh R. N. A systems biology approach uncovers the core gene regulatory network governing iridophore fate choice from the neural crest. PLoS Genet. 2018, 14 (10), e1007402 10.1371/journal.pgen.1007402. 30286071 PMC6191144

[ref35] Mahamid J.; Pfeffer S.; Schaffer M.; Villa E.; Danev R.; Cuellar L. K.; Forster F.; Hyman A. A.; Plitzko J. M.; Baumeister W. Visualizing the molecular sociology at the HeLa cell nuclear periphery. Science 2016, 351 (6276), 969–972. 10.1126/science.aad8857. 26917770

[ref36] Spiewak J. E.; Bain E. J.; Liu J.; Kou K.; Sturiale S. L.; Patterson L. B.; Diba P.; Eisen J. S.; Braasch I.; Ganz J.; Parichy D. M. Evolution of Endothelin signaling and diversification of adult pigment pattern in Danio fishes. PLoS Genet. 2018, 14 (9), e1007538 10.1371/journal.pgen.1007538. 30226839 PMC6161917

[ref37] Gur D.; Bain E. J.; Johnson K. R.; Aman A. J.; Pasoili H. A.; Flynn J. D.; Allen M. C.; Deheyn D. D.; Lee J. C.; Lippincott-Schwartz J.; Parichy D. M. In situ differentiation of iridophore crystallotypes underlies zebrafish stripe patterning. Nat. Commun. 2020, 11 (1), 1–14. 10.1038/s41467-020-20088-1. 33319779 PMC7738553

[ref38] Xue C.; Lin T. Y. W.; Chang D.; Guo Z. F. Thioflavin T as an amyloid dye: fibril quantification, optimal concentration and effect on aggregation. R. Soc. Open Sci. 2017, 4 (1), 160696 10.1098/rsos.160696. 28280572 PMC5319338

[ref39] Aggeli A.; Nyrkova I. A.; Bell M.; Harding R.; Carrick L.; McLeish T. C. B.; Semenov A. N.; Boden N. Hierarchical self-assembly of chiral rod-like molecules as a model for peptide beta-sheet tapes, ribbons, fibrils, and fibers. Proc. Natl. Acad. Sci. U. S. A. 2001, 98 (21), 11857–11862. 10.1073/pnas.191250198. 11592996 PMC59814

[ref40] Watt B.; van Niel G.; Raposo G.; Marks M. S. PMEL: a pigment cell-specific model for functional amyloid formation. Pigment Cell Melanoma Res. 2013, 26 (3), 300–15. 10.1111/pcmr.12067. 23350640 PMC3633693

[ref41] Hurbain I.; Geerts W. J.; Boudier T.; Marco S.; Verkleij A. J.; Marks M. S.; Raposo G. Electron tomography of early melanosomes: implications for melanogenesis and the generation of fibrillar amyloid sheets. Proc. Natl. Acad. Sci. U.S.A. 2008, 105 (50), 19726–31. 10.1073/pnas.0803488105. 19033461 PMC2604932

