# Supplementary material for: Plate-like Guanine Biocrystals Form via Templated Nucleation of Crystal Leaflets on Preassembled Scaffolds
Source: J Am Chem Soc. 2022 Dec 5;144(49):22440–5. doi: 10.1021/jacs.2c11136 (PMC9756333; doi:10.1021/jacs.2c11136)
Supplement: Supplementary file 4 — ja2c11136_si_004.pdf [file ja2c11136_si_004.pdf]

Supporting information

Plate-like guanine biocrystals form via templated nucleation of crystal leaflets on preassembled scaffolds

Zohar Eyal[1], Rachael Deis[1], Neta Varsano[2], Nili Dezorella[2], Katya Rechav[2], Lothar Houben[2], Dvir Gur[1*]

[1] Department of Molecular Genetics, Weizmann Institute of Science, Rehovot 76100, Israel.

[2] Department of Chemical Research Support, Weizmann Institute of Science, Rehovot 76100, Israel.

[*]Corresponding author. Email: Dvir.gur@weizmann.ac.il

Materials and Methods

Zebrafish Husbandry and Handling. Zebrafish (*Danio rerio*) were housed at ~28°C, 14 hr light:10 hr dark and fed with Artemia and flake food. Fish were maintained and fed following the standard protocols (Westerfield, 2000). The transgenic PNP4a line, Tg(*pnp4a:PALM-mCherry*), was maintained by crossing RFP positive males to RFP positive females and their progeny were subsequently screened for RFP by sorting embryos 3 days post-fertilization (dpf) for RFP signal using fluorescence microscopy.

Cell Dissociation and Isolation. Fish were anesthetized with Tricaine and immersed in TrypLE Express (Invitrogen, 12604039). Fish were incubated at 37°C and shaken at 200 rpm for 1 hr, followed by mechanical disruption with a Pasteur pipette to further dissociate the cells. Cells were then strained through a 40 µM cell strainer with HL-15 buffer and centrifuged (HL-15 Buffer contains: Hank's Balanced salt solution 40% (Sigma H8264) and 60% Leibovitz's L-15 Medium (Gibco 21083-027)). Dissociated cells were then pelleted at 3500 rcf for 5 minutes at 4°C, then resuspended 5 ml of fresh HL-15.

Live Imaging. Live imaging of cells was carried out on an inverted Zeiss LSM 900 using a Plan-Apochromat 63x/1.4 Oil DIC M27. Samples were illuminated with 561 nm and 405 nm laser lines. Cells were isolated via FACS and then incubated with 25 µM of Thioflavin-T (ThT) (Sigma, T3516) for 30 min prior to live imaging. After incubation with ThT cells were gently washed 2X in fresh HL-15 medium and plated on glass-bottom well imaging plates. 0.5% low melt agarose was then added to the plates to keep cells attached to the bottom for the duration of imaging.

Chemical fixation and conventional TEM. Samples were fixed with 4% paraformaldehyde, 2% glutaraldehyde in 0.1 M cacodylate buffer containing 5 mM $CaCl_2$ (pH 7.4) for 1 hr, postfixed in 1% osmium tetroxide supplemented with 0.5% potassium hexacyanoferrate trihydrate and potassium dichromate in 0.1 M cacodylate for 1 hr, stained with 2% uranyl acetate in double distilled water for 1 hr, dehydrated in graded ethanol solutions and embedded in epoxy resin. Ultrathin sections (70–90 nm) were obtained with a Leica EMUC7 ultramicrotome and transferred to Formvar Support film slot grids (EMS). Grids were stained with lead citrate and examined with a Tecnai SPIRIT transmission electron microscope (Thermo Fisher Scientific). Digital electron micrographs were acquired with a bottom-mounted Gatan OneView camera.

FACs Sorting. Cells were isolated from Tg(*pnp4a:PALM-mCherry*) positive fish cells and sorted via Fluorescence-Activated Cell Sorting (FACS). Following resuspension in 5 ml cold HL-15 with 1% BSA, the isolated cells were incubated with Hoechst to mark the nuclei for 30 min prior to FACs. Cells were analyzed and sorted using a BD FACSAria™ III Cell Sorter with a 100 µM nozzle. Cells were illuminated using a both 405 and 561 nm lasers. Cells were gated based on attributes to separate cells from each other as well as from cellular debris. Cellular debris were detected using forward, side scatter and Hoechst signals to select against the smallest particles (1 mm or less). Cells were additionally sorted and enriched based on detection using 561 nm filters, corresponding to the *pnp4a:PALM-mCherry* signal. Cells were collected into ice-cold HL-15 medium with 1% BSA and kept on ice until mounted on grids for downstream imaging.

High pressure freezing zebrafish larvae. 56, 72 and 96 hpf zebrafish larvae were anesthetized using Tricaine and decapitated. About 3 larvae were placed between two metal discs (2 mm

diameter; cavity, 0.2 mm) in 10% dextran solution and cryo-immobilized using a Leica LM ICE high pressure freezing device (Leica Microsystems, Germany).

Freeze fracture, cryo-SEM. The high-pressure frozen zebrafish larvae samples were shuttled using a vacuum cryo-transfer device (VCT 100, Leica Microsystems, Germany). The sample was transferred into a freeze-etching/freeze-fracture device (BAF 60, Bal-Tec, Germany), the stage of which was maintained at -120°C and a vacuum of about 5×10^{-7} mbar. After fracturing, the disc remaining in the sample holder was coated with 6 nm of platinum. The coated sample was transferred to the SEM (Ultra 55, Zeiss, Germany) and observed at −120°C and a vacuum of about 5×10^{-7} mbar, using an acceleration voltage of 1 kV, an aperture size of 10 µm, and a working distance of 2 mm.

Cryo FIB-SEM. High-pressure frozen larvae samples were fractured as done for the cryo SEM, except the temperature was at -160°C. The coated sample was transferred to cryo FIB-SEM (Crossbeam 550, Zeiss, Germany) using a vacuum cryo-transfer device (VCT 100, Leica Microsystems, Germany). At all times, the temperature of the cryo-stage was below −150°C, and the vacuum inside the chamber of was around 5×10^{-7} mbar. Rough milling was done prior to imaging (in order to expose the region of interest) at 30 kV FIB acceleration voltage and 1.5 nA FIB probe current. Fine milling was done with the following milling parameters: 5 mm working distance, 30 kV FIB acceleration voltage and between 100 to 700 pA FIB probe current. A total of 5 data sets from different samples (56, 72 and 96 hpf frozen larvae) were milled that had dimensions of 30-65 µm × 8.3-25 mm with slice thickness of 10 or 20 nm. Imaging was done using the following parameters: lateral image pixel size between 6 and 19.5 nm, field of view 12-40 µm width and 2 kV SEM acceleration voltage.

Image processing of 3D data was performed as follows: To correct for the vertical stripes occurring in the stacks, we applied the wavelet decomposition algorithm as published in Spehner D. et al.[1]. We've used the coif wavelet of N = 10 and vertical coefficient σ = 10. This approach was chosen as it preserves the overall intensity of the image better than with a simple Fourier filtering approach. The images were then corrected for local charge imbalance using the morphological reconstruction (dilation) approach[1]. The algorithm was implemented using python 3.7. The images were automatically aligned with the MIB software (Microscopy Image Browser, University of Helsinki Institute of Biotechnology EM unit)[2]. The aligned stack was further aligned manually using the Amira3D (Thermo Scientific) alignment tool.

CryoET. Sorted iridophores cells (3.5 µL) with 15 nm gold beads (1 µL) were applied to glow-discharged holey carbon R2/2 Cu 200 SiO_2 mesh grids (Quantifoil) coated with collagen, Type I, Rat Tail (EMD Millipore 08-115) for cell adherence. The grids were blotted and vitrified by plunging into liquid ethane using a Leica EM GP automatic plunger, under 4°C and 90% humidity conditions. Frozen grids were kept in liquid nitrogen until used. Data was collected on a Titan Krios TEM G3i (Thermo Fisher Scientific) equipped with a BioQuantum energy filter with a K3 direct electron detector (Gatan Inc.). Data sets were collected at 300 kV with the K3 camera (counting mode) using SerialEM software[3]. The TEM magnification corresponded to a camera pixel size of 1.6 Å, and the target defocus was set to 3 µm. The total dose for a full tilt series was 120 electrons per $Å^2$. Tomograms tilt series were collected using the dose-symmetric scheme, ±60° at 2° degrees steps. The tilt series images alignment and reconstruction were performed in IMOD[4].

Cryo 4D STEM. Scanning transmission electron microscope (STEM) images and analytical EDS maps were acquired in a double aberration-corrected Themis-Z microscope (Thermo Fisher Scientific Electron Microscopy Solutions, Hillsboro, USA, (TFS)) at an accelerating voltage of 200 kV. All measurements were completed on fully vitrified plunge-frozen samples in a Gatan 914 cryo-transfer holder. STEM images were recorded using a Fischione Model 3000 detector and a TFS BF detector. EDS hyperspectral data was obtained with a Super-X SDD detector and quantified with the Velox software (TFS) through background subtraction and spectrum deconvolution.

4D STEM[5] datasets were obtained on the EMPAD detector (Electron Microscope Pixel Array Detector)[6] that allowed rapid data collection of unsaturated diffraction patterns with single electron sensitivity. An electron probe with a convergence angle of 0.2 mrad was adjusted and further defocussed by typically 5-10 µm to increase the real space probe size to several 10 nm in diameter and to reduce the electron fluence. The defocussed electron beam was rastered without beam overlap across the sample. A primary beam current between 1 pA resulted in a total fluence of less than 1 $e^-/Å^2$ per exposure frame time of 2 ms, i.e., per beam position. All 4D STEM datasets were analyzed with custom-written software.

Segmentation- Segmentation and 3D representation of the reconstructed data was done using Amira® software (Thermo Scientific). Data segmentation was performed based on contrast variations following the unique shape and structure of each component.

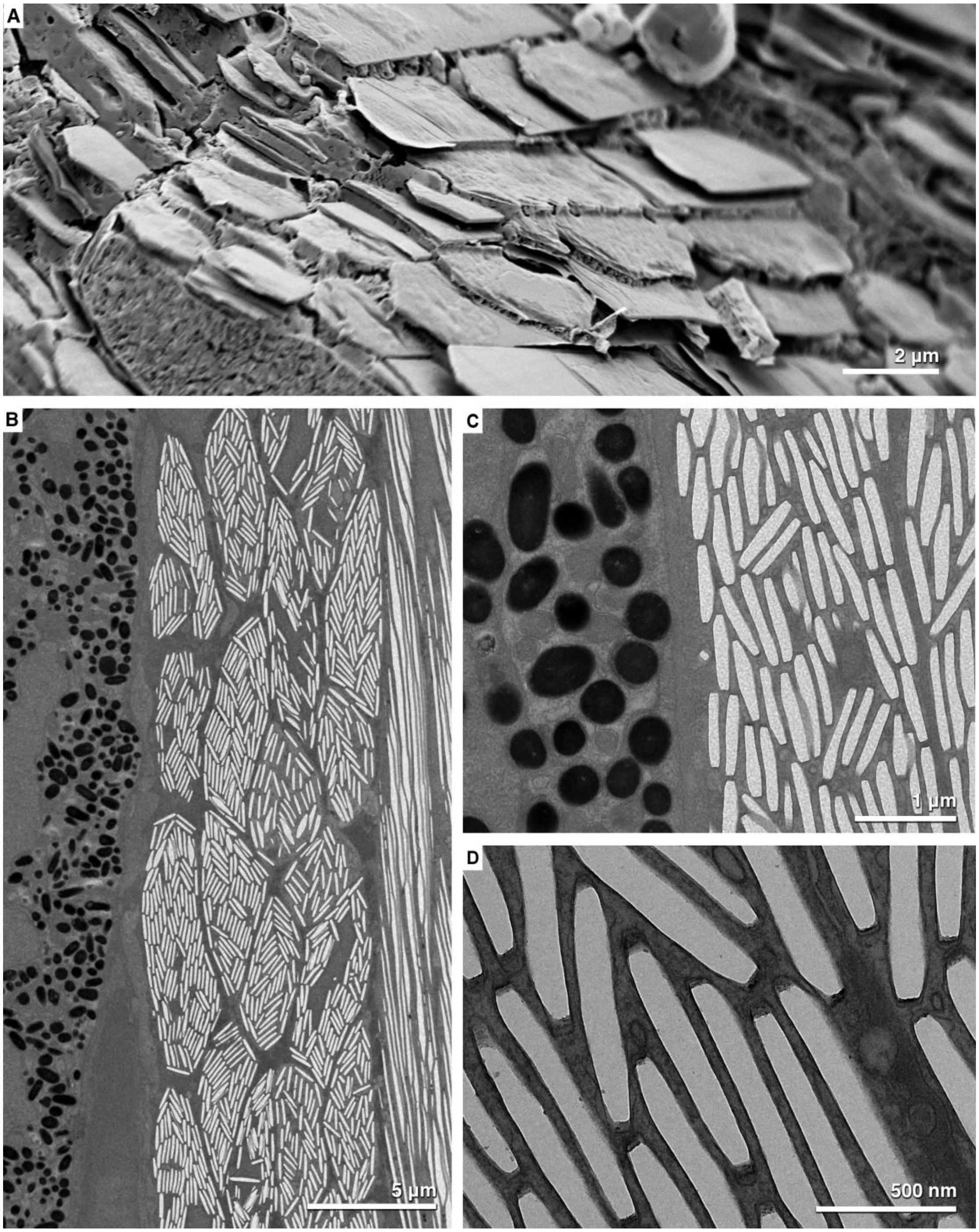

Figure S1: Iridophores in adult zebrafish eye. (A) Cryo SEM image showing stacks of mature guanine crystals. **(B-D)** Conventional TEM images of thin slices of an adult zebrafish eye.

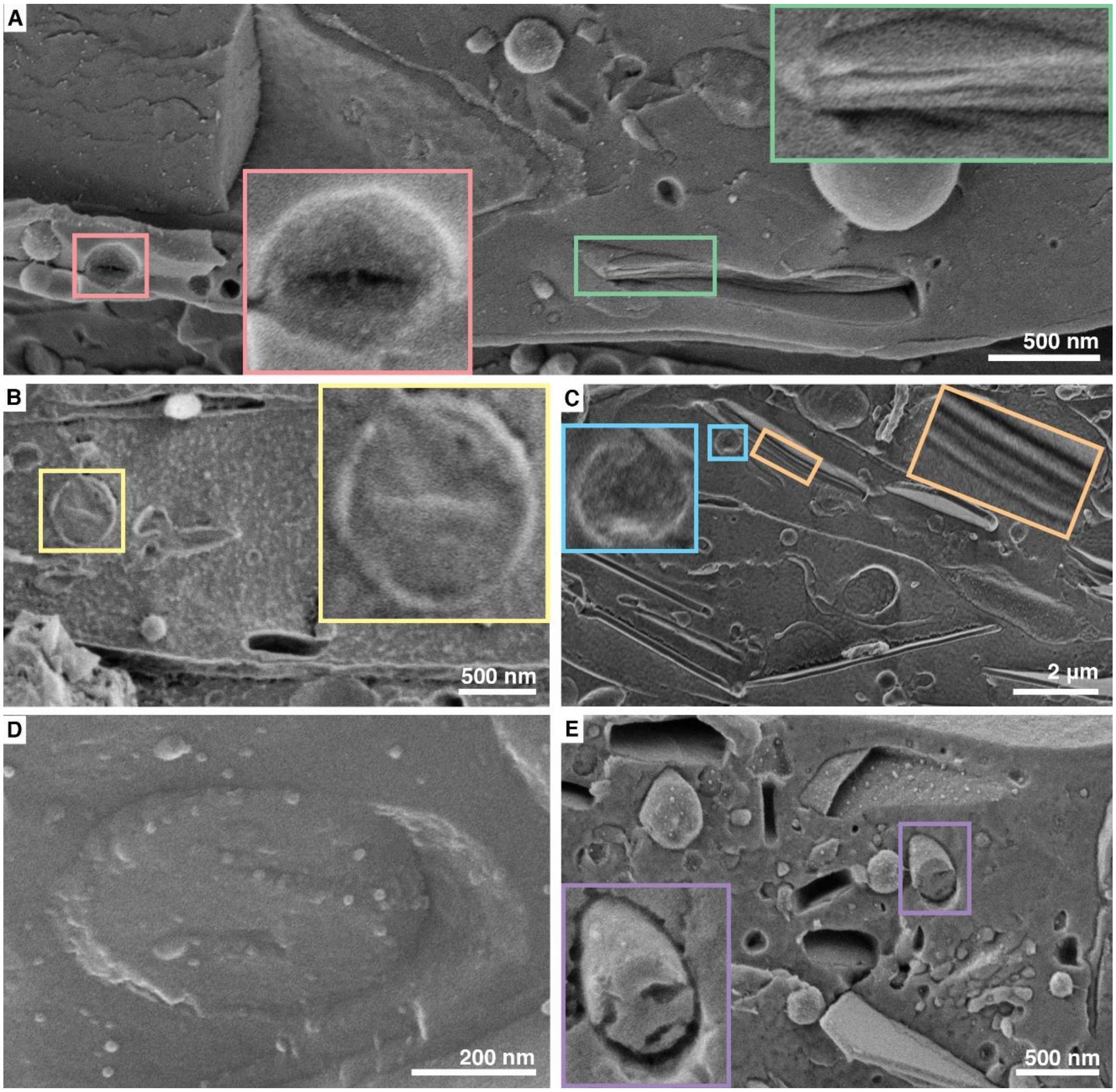

Figure S2: Early crystal forming iridosomes. (A-E) Cryo SEM images of young iridosomes. (A) An iridosome with a hole where a crystal used to be (pink) next to a more mature iridosome with several crystal leaflets (green). (B) an early iridosome containing a single leaflet (yellow). (C) An iridosome with a single leaflet (blue), next to a more mature iridosome with several crystal leaflets (orange). (D) an early iridosome with two very small crystal leaflets. (E) An iridosome with several holes where the thin leaflets were located (purple), next to a mature crystal.

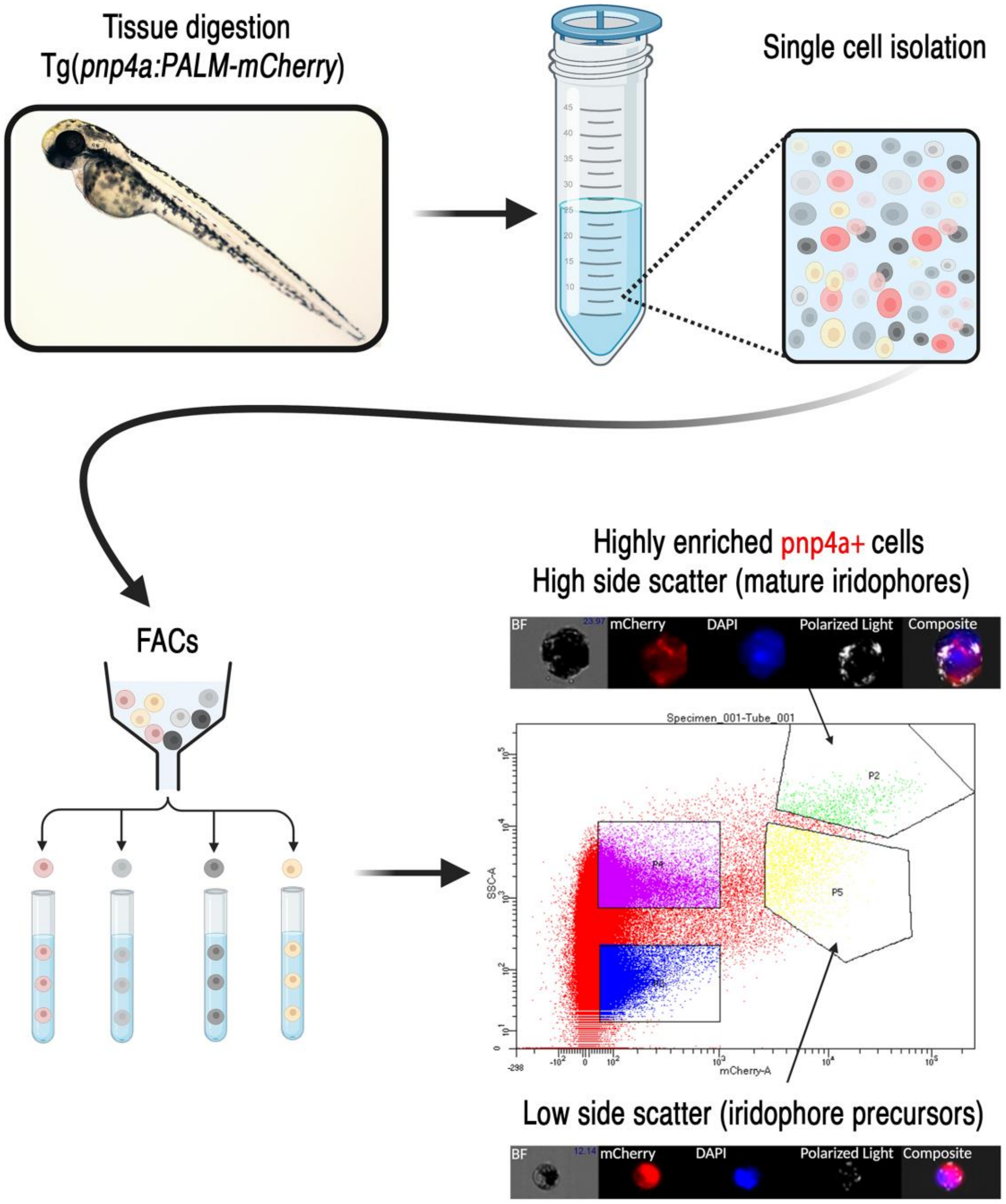

Figure S3: FACS workflow for separating iridophores from whole zebrafish larvae. Schematic overview of zebrafish larvae digestion and cell isolation via Fluorescence-Activated Cell Sorting (FACS). Cells were isolated from Tg(*pnp4a:PALM-mCherry*) positive fish mix. After cell isolation, cells were analyzed and sorted using a BD FACSAria™ III Cell Sorter and were gated based on attributes to separate cells from each other as well as from cellular debris. Cellular debris were detected using forward, side scatter and Hoechst signals to select against the smallest particles (1 mm or less). Cells were additionally sorted and enriched based on detection using 561 nm filters, corresponding to the *pnp4a:PALM-mCherry* signal. Different populations of iridophores can be distinguished using this method based on their mCherry signal in combination with the intensity of their side scatter.

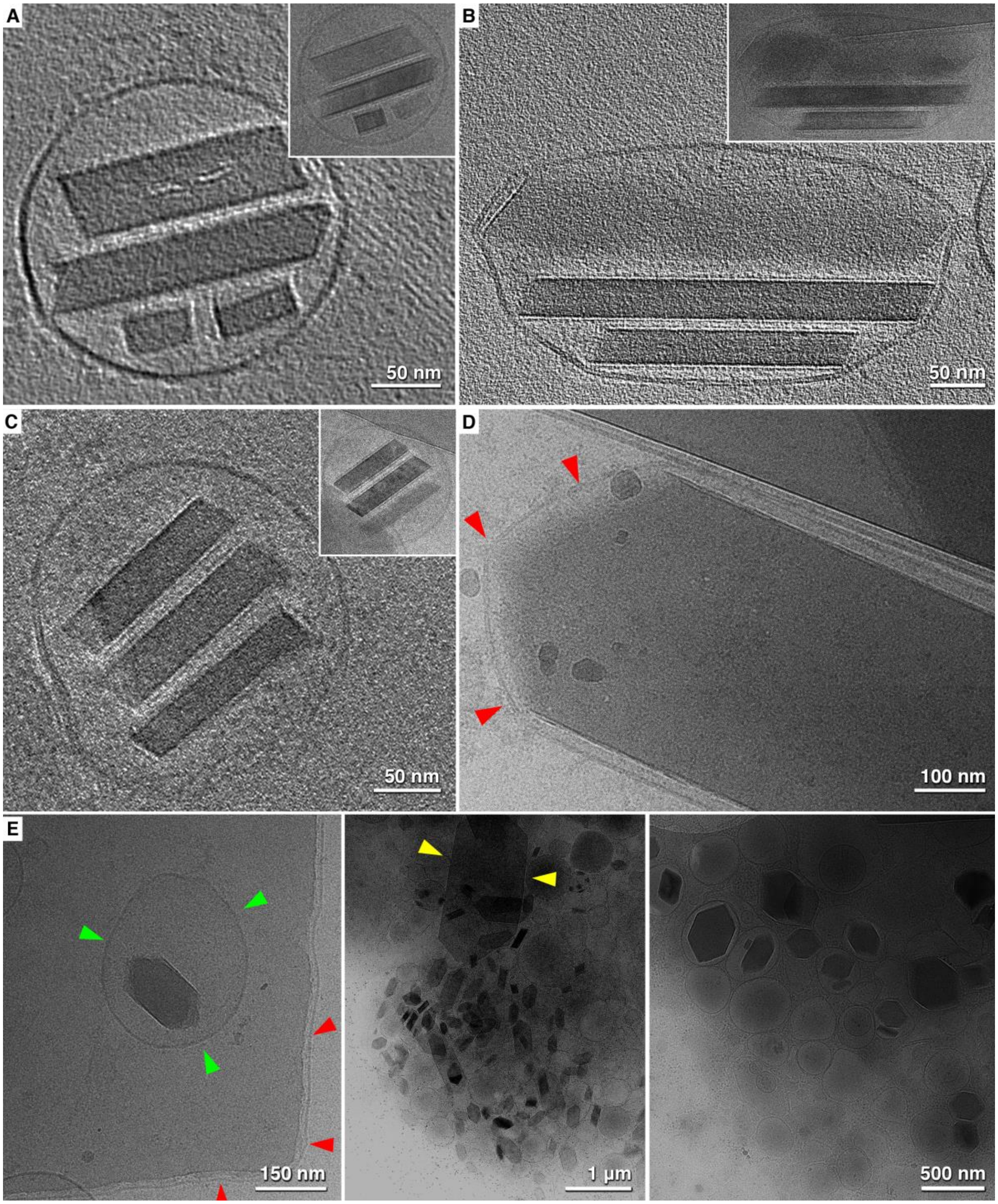

Figure S4: Cryo TEM images of iridosomes from of isolated cells of zebrafish larvae after FACS. **(A-C)** CryoET reconstructions of early iridosomes with several crystal leaflets. Insets show the 2D projections of the same iridosome. (**D-E**) CryoET 2D projections of a mature iridosome, where the membrane tightly engulfs the crystal (red arrowheads). In (E) An early iridosome containing a crystal with considerable distance from the delimiting membrane (green arrowheads) is shown on top of the mature crystal. (**F-G**) CryoET 2D projections of iridophores from 72 hpf (F) and 48 hpf (G), showing iridosomes at different maturation stages.

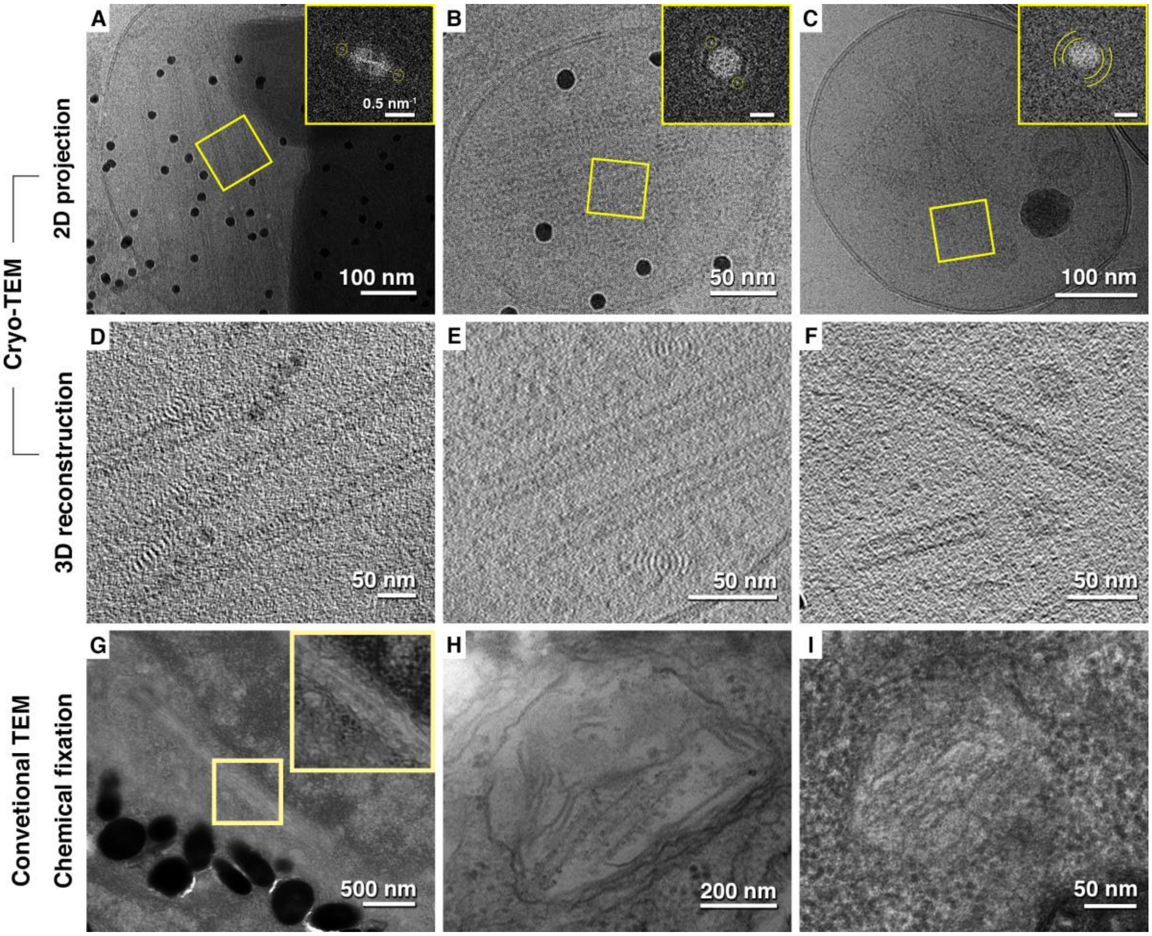

Figure S5: TEM imaging of fibers inside iridosomes. (A-C) Cryo TEM projection images of early, fibers containing iridosomes. Insets show FFT taken from the areas marked by yellow squares in the projection images. The fibers had a periodicity of ~1.9 nm, which correspond to the inter ribbon spacings of the beta sheet fibril**. (D-F)** CryoET reconstructions of the fibers shown in the 2D projections. The fibers thickness was on average 19.6±0.53 nm (n=49, standard error), and their length was on average 308.2 ±21.9 nm (n=27, standard error). **(G-I)** Conventional TEM images of thin slices taken from zebrafish larvae eyes showing fiber containing iridosomes.

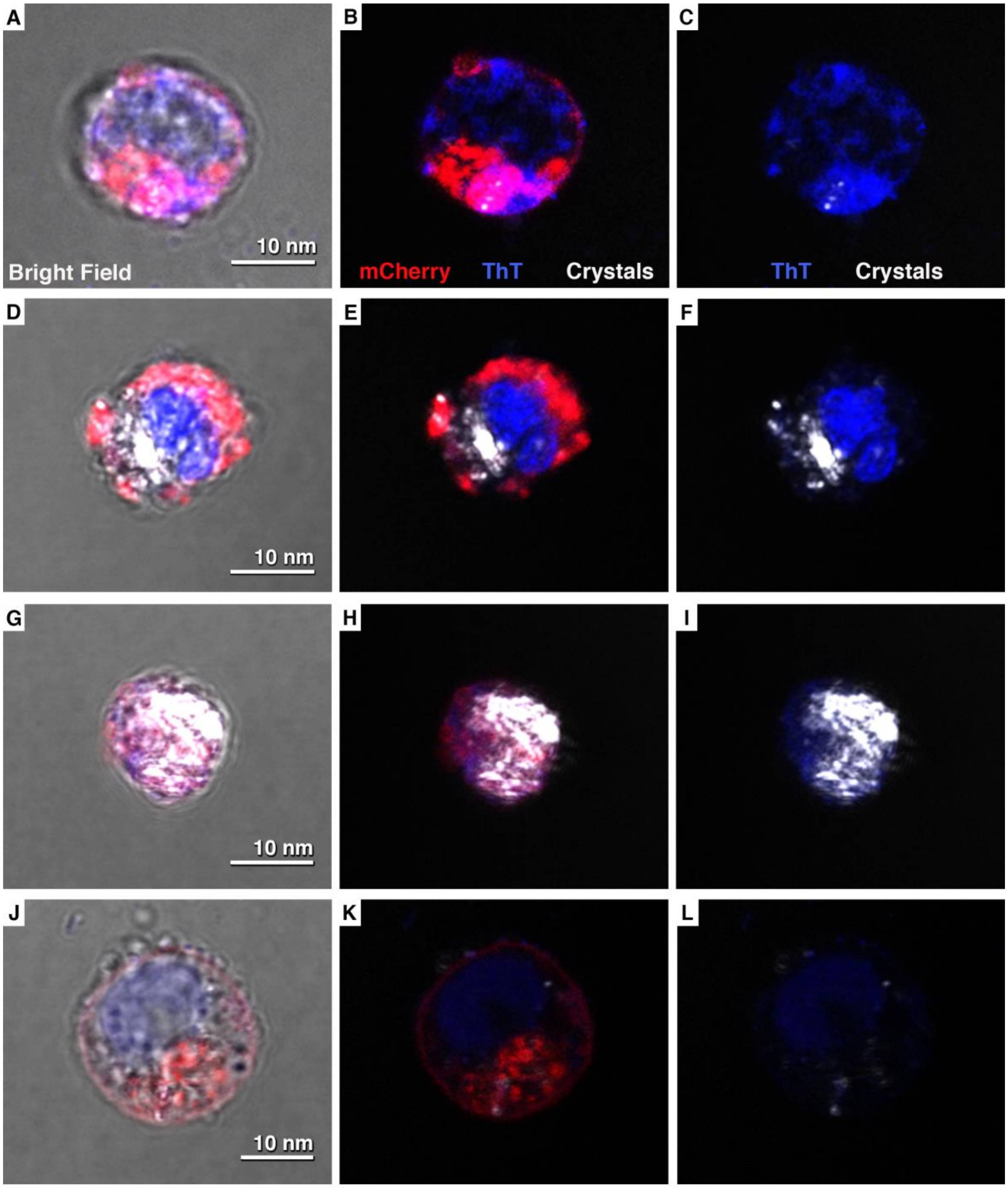

Figure S6: Thioflavin-T (ThT) live imaging reveals amyloid proteins. (**A-L**) Different stages of iridophores were isolated after FACS and incubated ThT prior to confocal live-cell imaging. Isolated iridophores express mCherry, and contain crystals of different sizes. Strong ThT signal can be found in early stage iridophores that express high mCherry, but have relatively small amounts of crystals (A-F). However, the amount of ThT signal detected in cells was dependent on the amount of crystals. More mature iridophores that were packed full of crystals (G-I) had little to no ThT signal. (J-L) A cell expressing mCherry with only background ThT signal for comparison.

References

(1) Spehner, D., Steyer, A.M., Bertinetti, L., Orlov, I., Benoit, L., Pernet-Gallay, K., Schertel, A. and Schultz, P. Cryo-FIB-SEM as a promising tool for localizing proteins in 3D. *Journal of structural biology* **2020**, *211*(1), p.107528.

(2) Belevich, I., Joensuu, M., Kumar, D., Vihinen, H. and Jokitalo, E. Microscopy image browser: a platform for segmentation and analysis of multidimensional datasets. *PLoS biology* **2016**, *14*(1), p.e1002340.

(3) Mastronarde, D.N. Automated electron microscope tomography using robust prediction of specimen movements. *Journal of structural biology* **2005**, *152*(1), pp.36-51.

(4) Kremer, J.R., Mastronarde, D.N. and McIntosh, J.R. Computer visualization of three-dimensional image data using IMOD. *Journal of structural biology* **1996**, *116*(1), pp.71-76.

(5) Ophus, C. Four-dimensional scanning transmission electron microscopy (4D-STEM): From scanning nanodiffraction to ptychography and beyond. *Microscopy and Microanalysis* **2019**, *25*(3), pp.563-582.

(6) Tate, M.W., Purohit, P., Chamberlain, D., Nguyen, K.X., Hovden, R., Chang, C.S., Deb, P., Turgut, E., Heron, J.T., Schlom, D.G. and Ralph, D.C. High dynamic range pixel array detector for scanning transmission electron microscopy. *Microscopy and Microanalysis* **2016**, *22*(1), pp.237-249.
